# Author Correction: Zinc isotopes from archaeological bones provide reliable trophic level information for marine mammals

**DOI:** 10.1038/s42003-021-02343-3

**Published:** 2021-06-23

**Authors:** Jeremy McCormack, Paul Szpak, Nicolas Bourgon, Michael Richards, Corrie Hyland, Pauline Méjean, Jean-Jacques Hublin, Klervia Jaouen

**Affiliations:** 1 Department of Human Evolution, Max Planck Institute for Evolutionary Anthropology, Leipzig, Germany; 2 Department of Anthropology, Trent University, Peterborough, ON Canada; 3 Applied and Analytical Paleontology, Institute for Geosciences, Johannes Gutenberg-University Mainz, Mainz, Germany; 4 Department of Archaeology, Simon Fraser University, Burnaby, BC Canada; 5 Géosciences Environnement Toulouse, UMR 5563, CNRS, Observatoire Midi Pyrénées, Toulouse, France

**Keywords:** Biogeography, Stable isotope analysis, Marine mammals, Element cycles, Palaeoecology

Correction to: *Communications Biology* 10.1038/s42003-021-02212-z, published online 3 June 2021.

In the original version of the Article, the title was incorrectly stated as “Zinc isotopes from archaeological bones provide reliable tropic level information for marine mammals.” This has now been corrected to “Zinc isotopes from archaeological bones provide reliable trophic level information for marine mammals” in the PDF and HTML versions of the article.

