# Supplementary material for: Zinc isotopes from archaeological bones provide reliable trophic level information for marine mammals
Source: Commun Biol. 2021 Jun 3;4:683. doi: 10.1038/s42003-021-02212-z (PMC8175341; doi:10.1038/s42003-021-02212-z)
Supplement: Supplementary file 2 — Supplementary Information [file 42003_2021_2212_MOESM2_ESM.pdf]

Supplementary Information for:

Zinc isotopes from archaeological bones provide reliable tropic level information for marine mammals

Jeremy McCormack, Paul Szpak, Nicolas Bourgon, Michael Richards, Corrie Hyland, Pauline Méjean, Jean-Jacques Hublin & Klervia Jaouen

This PDF file contains:

1. Supplementary Note
    1.1 *P. hispida* and *U. maritimus* diet
    1.2 Archaeological context
2. Supplementary Methods
    2.1 Mineral dissolution experiment
    2.2 Stable carbon and nitrogen isotope analyses
3. Supplementary Discussion
    3.1 Preservation of $\delta^{15}N$, $\delta^{13}C$ and $\delta^{66}Zn$ values
    3.2 Impact of collagen on bone $\delta^{66}Zn$ values and application for multi-proxy dietary studies
    3.3 Baseline carbon and nitrogen isotope variability recorded in *P. hispida* bones
    3.4 Trophic level assessment

Supplementary Figures 1-9

Supplementary Tables 1-6

Supplementary References

1. Supplementary Note

1.1 *P. hispida* and *U. maritimus* diet

Pusa hispida is not a highly specialised feeder and its diet can vary seasonally and geographically, and includes teleosts, amphipods and other crustaceans and cephalopods[1,2,3]. Its main food source is Arctic cod (*Boreogadus saida*) and other gadids for most Arctic regions[3,4,5]. Based on stomach content analysis, perfluoroalkyl compounds (PFCs), $\delta^{15}N$ and $\delta^{13}C$ tissue values across the Arctic, the diet of different *P. hispida* populations is known to vary locally and seasonally to some degree[5,6,7]. Still, modern *P. hispida* populations are thought to inhabit the same trophic level across the Arctic[6].

Pusa hispida is the main food source for *U. maritimus* in the Arctic. However, modern polar bears are not a single cosmopolitan population[8] and the contribution of *P. hispida* to a bear's diet relative to other prey species (e.g., *Erignathus barbatus, Pagophilus groenlandicus, Phoca vitulina, Odobenus rosmarus, Delphinapterus leucas*) varies within different populations[9,10]. Additionally, *U. maritimus* may also scavenge the remains of larger whale species when available[11]. A contribution of terrestrial food to *U. maritimus* diet is negligible[12].

1.2 Archaeological context

The materials analysed are derived primarily from occupations associated with the Thule Inuit, with a few exceptions. All bones were identified based on skeletal morphology. Additionally, terrestrial mammal bones were identified as *U. maritimus* based on skeletal morphology and $\delta^{15}N$ values, which are distinct to other terrestrial mammals that could be present in the assemblages. All sites have been dated by AMS ^{14}C as outlined in Supplementary Table 3 in which references are provided for publications providing additional details about these sites. For simplicity, when analysing geographic variability, the following sites were grouped together as a single location: OkRn-1 with OlRr-1, KTZ-304 with KTZ-087, PaJs-13 with PcJq-5 and PeJr-1.

2. Supplementary Methods

2.1 Mineral dissolution experiment

Bone samples and reference materials NIST SRM 1400 and NIST SRM 1486 were subjected to different dissolution methods to investigate the impact of the organic bone phases on the bulk bone Zn isotope signal (Supplementary Figure 2). For that purpose, we resampled 26 *P. hispida* bones to measure $\delta^{66}Zn$ of the bulk bone, the mineral phase and the collagen phase of the same bone material. The column chromatography steps (3.1.2) for a quantitative recovery of sample Zn were the same for all samples regardless of the dissolution methods used.

Method 1: Mineral phase dissolution only

Samples were transferred into acid-cleaned 2 ml polypropylene microcentrifuge tubes and demineralised in 1 ml 1 M hydrochloric acid (HCl) at room temperature. After two days of digestion, the demineralisation progress was checked with a glass pipette. If the samples were still hard, the solution was extracted after centrifugation, and another 1 ml HCl added to the residue containing tube. After another two days, the

samples were checked again. For all samples, the collagen residue was soft and spongy no later than after four days, indicating complete demineralisation. The tubes were then centrifuged and the solution (dissolved mineral phase) was extracted for Zn isotope analyses. The remaining insoluble collagen was also collected (with 1 ml ultrapure water added). The dissolved mineral phase was evaporated in open (Savillex) perfluoroalkoxy (PFA) vials on a hotplate for 5 h at 120 °C, then re-dissolved in 1 ml hydrobromic acid (HBr, 1.5 M) and subsequently placed in an ultrasonic bath for 30 min.

Collagen samples for δ^{66}Zn analyses were rinsed with ultrapure water, centrifuged, and rinsed again. Samples were then dried down and dissolved with 1 ml ultrapure (65 %) concentrated nitric acid (HNO_3) for 1 h at room temperature followed by 1 h in a closed vial on the hotplate at 120 °C. Finally, samples were dried down for 5 h at 120 °C and re-dissolved in 1 ml 1.5 M HBr and ultrasonicated for 30 min.

Method 2: Bone dissolution following enamel dissolution protocol

Bone samples were dissolved following an established protocol primarily applied to enamel samples[13,14]. Samples were dissolved in closed PFA vials with 1 ml 1 M HCl on a hotplate for 3 h at 120 °C and then evaporated at 120 °C. The residue was then dissolved in 1 ml 1.5 M HBr and placed in an ultrasonic bath for 30 min.

Method 3: Bulk bone dissolution

Bone samples were dissolved with 1 ml ultrapure (65 %) concentrated HNO_3 for 1 h at room temperature in an open PFA vial, followed by 1 h in a closed vial, on a hotplate, at 120 °C. Samples were then dried down at 120 °C, re-dissolved in 1 ml 1.5 M HBr, and ultrasonicated for 30 min.

2.2 Stable carbon and nitrogen isotope analyses

Carbon and nitrogen isotope and elemental compositions were determined using an IsoPrime continuous flow isotope-ratio mass spectrometer (CF-IRMS) coupled to a Vario Micro elemental analyser (Elementar, Hanau, Germany) at the University of British Columbia. Sample measurements were calibrated relative to VPDB (δ^{13}C) and AIR (δ^{15}N) using USGS40 and USGS41[15]. The standard deviations and number of calibration (quality control) standards used in all of the analytical sessions are listed in Supplementary Table 4.

Standards used to monitor accuracy and precision are listed in Supplementary Table 5. The isotopic compositions used as the accepted values for these internal standards represent long-term averages. Supplementary Table 6 summarises the mean and standard deviation of carbon and nitrogen isotope compositions for all check (quality assurance) standards analysed alongside the samples presented in this study. All of the samples were analysed in at least duplicate. One internal standard (SUBC-1, seal bone collagen) was in the process of attaining an average long-term value, so we treated this as a sample replicate rather than a QA standard (155 aliquots of this material were analysed alongside these samples). The pooled standard deviations for the sample replicates were ±0.12 ‰ for δ^{13}C and ±0.14 ‰ for δ^{15}N (*df*=300).

Standard uncertainty for the δ^{13}C and δ^{15}N measurements of the samples was estimated following Szpak et al.[16], which largely follows the method presented in Magnusson et al.[17]. Systematic errors ($u_{(bias)}$) were calculated to be ±0.08 ‰ for δ^{13}C and ±0.12 for δ^{15}N based on the known uncertainty in the check standards and the observed standard deviations of those check standards from the known values.

Random errors ($uR_{(w)}$) were calculated to be ±0.14 ‰ for $\delta^{13}C$ and ±0.17 ‰ for $\delta^{15}N$ based on the pooled standard deviations of the check standards and sample replicates. Standard uncertainty calculated as the root-sum-square of $u_{(bias)}$ and ($uR_{(w)}$) was determined to be ±0.16 for $\delta^{13}C$ and ±0.21 for $\delta^{15}N$.

3. Supplementary Discussion

3.1 Preservation of $\delta^{15}N$, $\delta^{13}C$ and $\delta^{66}Zn$ values

All bone samples demonstrate exceptional bone collagen preservation based on collagen yields and elemental compositions (wt% C, wt% N, C:N ratios) within the range of modern mammal bone[19,20] (Supplementary Data 1). Diagenetic modification of original bone $\delta^{66}Zn$ values may be expected following bone recrystallisation and associated accumulation or leaching of trace elements or secondary mineral precipitation. However, such diagenetic modifications are strongly associated with the loss of the organic matrix causing increased porosity and bioapatite recrystallisation[21,22]. The excellent preservation of the collagen argues against significant diagenetic modification of the bioapatite Zn content. Reynard and Balter[23] suggested that diagenetic modification of trace element content might result in a correlation between concentration, expressed as 1/concentration, and isotopic composition of the element in question. We observe no correlation between $\delta^{66}Zn$ and Zn concentrations when analysing all *P. hispida* or *U. maritimus* samples (R^2= 1.38e-4, p = 0.97, R^2= 1.20e-2, p = 0.46, Supplementary Figure 1). The lack of a correlation suggests that soil Zn addition and/or diagenetic zinc incorporation into the bone samples did not contribute to the samples' $\delta^{66}Zn$ value. Correlation of *P. hispida* zinc concentration and $\delta^{66}Zn$ values within a single site is also typically weak or non-existent. However, for the KkJg-1 and JfEl-4 sites there seems to be a statistically significant correlation between $\delta^{66}Zn$ and 1/[Zn], with R^2 of 0.44 (n=11, p = 0.03) and 0.53 (n=9, p = 0.01). Still, post-hoc Tukey pair-wise comparisons show that $\delta^{66}Zn$ values from both sites are not distinct from other sites in regards to their $\delta^{66}Zn$ values. Mean $\delta^{66}Zn$ values from the JfEl-4 site are the same as the mean values from the nearby KkDo-1 site (Figure 2), which does not show a correlation between Zn isotope composition and concentration. *P hispida* bones from both the JfEl-1 and KkDo-1 sites have, as with $\delta^{66}Zn$, very similar $\delta^{15}N$ and $\delta^{13}C$ values, distinct from other geographic regions (Figure 2, Supplementary Data 1), indicating preservation of original isotopic signals. Most importantly, for the JfEl-4 site *U. maritimus* samples show no correlation between $\delta^{66}Zn$ and 1/[Zn] (R^2 = 0.06, n = 5, p = 0.68) arguing against a diagenetic alteration or contamination causing the correlation for *P. hispida* for this site. Instead of diagenetic modification or soil contamination, individual taxonomically misidentified bones could also explain correlations between proxies (including [Zn]). Alternatively, these populations may include individuals with higher mobility or differences in diet.

Surface water dissolved-Zn concentrations and isotopic compositions are expected to vary within the surface water across the Arctic to some degree as observed in other oceans[24,25]. Mean site Zn concentration and $\delta^{66}Zn$ values may therefore reflect variations in POM Zn concentration and isotopic composition passed along the food chain. Indeed, Zn concentrations in phytoplankton vary depending on Zn availability and primary producers[26]. In the Western Arctic for example, Zn:C stoichiometries for shelf phytoplankton were higher compared to offshore phytoplankton[27]. A weak correlation between $\delta^{66}Zn$ and Zn concentrations within a population may arise from individuals that are more mobile or distinct in their diet. In any case, a much higher correlation would be expected between Zn concentration and isotopic composition if diagenetic Zn modification or soil contamination would be a dominant influence for the

KkJg-1 and JfEl-4 sites. For example, more porous cancellous bone of *O. rosmarus* from the QjJx-1 site showed a much higher correlation ($R^2 = 0.82$) between 1/[Zn] and δ^{66}Zn values, likely due to the cancellous bone retaining soil particles[18].

For seal bone samples from the three sites JfEl-4, KcFs-2 and NkRi-3, taxonomic misidentification cannot be completely ruled out, i.e., some samples may also belong to other Phocidae than *P. hispida*. Additionally, the JfEl-4 site has, besides the QjJx-1 site, the highest on-site *P. hispida* bone δ^{15}N and δ^{66}Zn variability with 1.77 and 0.36 ‰, respectively. This could indicate that individual bones indeed belong to other Phocidae. Again, these sites generally have mean δ^{15}N, δ^{13}C and δ^{66}Zn values similar to other sites in the same geographic region (Figure 2, Supplementary Data 1). The three sites JfEl-4, KcFs-2, NkRi-3 do not belong to the populations drawn out to be distinct from others by δ^{66}Zn post-hoc Tukey pair-wise comparisons. If several bones from these sites would belong to different Phocidae, we may expect a higher isotopic variability for the sites, as some Phocidae were shown to have distinct δ^{15}N, δ^{13}C and δ^{66}Zn values due to differences in their diet[18]. Most importantly, however, *P. hispida* is by far the dominant faunal component in Arctic archaeological sites[28]. We assume that most, if not all, Phocidae bone samples from these sites, indeed belong to *P. hispida*.

The typically high homogeneity in *P. hispida* and *U. maritimus* bone δ^{15}N, δ^{13}C and δ^{66}Zn values within a site (Supplementary Data 1) strongly argues against sample diagenesis, contamination issues and/or taxonomic misidentifications as a significant cause of isotopic variability within and between sites. However, it may explain unusual *P. hispida* outlier δ^{15}N and δ^{66}Zn values from the QjJx-1 site on Little Cornwallis Island[18]. Even excluding the one *P. hispida* sample from the QjJx-1 site with an unusually high δ^{66}Zn value (1 ‰), post-hoc Tukey pair-wise comparisons draw out this population as distinct from others (Supplementary Figure 4). This population also has the highest variability of *P. hispida* bone collagen δ^{15}N values (3.85 ‰) and a high variability in δ^{13}C values (2.47 ‰). It is also possible that this variation originates from more mobile individuals within the population, or larger differences in food sources for individuals. Within a *P. hispida* population from Svalbard, Norway, Lone et al.[29] demonstrated, that 18 from 60 tagged individuals undertook extensive seasonal migrations. Individuals that are more mobile might consume a different type of prey in different regions, or prey for which tissue isotopic composition is influenced by different baseline values. If the samples from one site contain a higher percentage of bones from more mobile individuals, that population may have a higher δ^{15}N, δ^{13}C and δ^{66}Zn variability compared to others and perhaps demonstrate a correlation between different dietary proxies. For the KkJg-1 site in Hudson Bay, two *U. maritimus* samples show anomalously high δ^{66}Zn values which may relate to non-dietary factors such as contamination, misidentification, diagenesis or physiological effects. One of these two outlier samples has a distinct dark pervasive colouration of the bone, which may imply a contamination and/or preservation issues. A bone sample identified as *D. leucas* from the JfEl-4 site has an unusually low δ^{15}N value (11.82 ‰). We have too little *D. leucas* δ^{66}Zn values to draw a conclusion on its isotopic range. However, one *Odobenus rosmarus* bone from the same site has a similar δ^{15}N and δ^{13}C isotope composition and an only slightly higher δ^{66}Zn value (Supplementary Figure 9). We cannot exclude the possibility of taxonomic misidentification for the unusual *D. leucas* sample and have hence excluded it from further discussion. Still, a significant influence of diagenesis, soil contamination or taxonomical misidentification can be excluded for most samples.

3.2 Impact of collagen on bone δ^{66}Zn values and application for multi-proxy dietary studies

The treatment with different dissolution methods did not lead to any variation in the bone ash NIST SRM 1400 nor in the bone meal NIST SRM 1486 δ^{66}Zn values, despite the latter still containing a collagen organic component (Supplementary Figure 3). As with the SRM reference materials, the demineralisation methods tested herein did not lead to systematic significant variability in δ^{66}Zn values of selected samples (Supplementary Data 2). Although we used archaeological bone samples for this study, their collagen content was still as high as in modern mammal bones (Supplementary Data 1). Minor variability between bone samples treated with different dissolution methods may arise from resampling of larger bone fragments and potential heterogeneities within a larger bone sample. While each method fully demineralised the bone, the extent of collagen dissolution varied: from collagen preservation to complete collagen dissolution (Supplementary Figure 2). After collagen extraction following the dissolution protocol 1, we tried to measure the Zn isotopic composition of the collagen relative to the mineral phase. However, demineralised collagen Zn concentrations were between 0.08 and 1.2 µg/g bone, and therefore too low for zinc isotope analyses. As such, Zn bonded to the organic phase in bones likely has no impact on bulk bone Zn isotope compositions. It is possible that all Zn initially bonded to the organic phases of the bone may have been released during demineralisation regardless of the method used. However, the Zn concentration of the organic phase is likely too insignificant in comparison to that of the mineral phase. Zn^{2+} substitutes for Ca^{2+} in bioapatite[30] and synthesised hydroxylapatites[31]. The bone mineral phase acts as a sink for Zn, whereas Zn bound to the organic matrix appears to be volumetrically negligible compared to the bulk bone Zn[32].

The absence of an impact of collagen on bulk bone δ^{66}Zn values has some important implications for the use of Zn in bone as a dietary proxy. Fossil bone samples can be treated like modern samples, independent of the collagen preservation, provided they show neither Zn detrital contamination nor diagenetic modification. Mineral phase δ^{66}Zn can be coupled with collagen extraction protocols applied for δ^{15}N and δ^{13}C analyses on the same sample. Dissolution method 1 followed a protocol similar to common collagen extraction protocols applied for bone collagen δ^{13}C and δ^{15}N analysis. Thus, δ^{66}Zn analysis could be routinely coupled with δ^{15}N and δ^{13}C on a single sample. Coupling of δ^{66}Zn with δ^{15}N and δ^{13}C analyses will allow a more robust, complementary multi-proxy dietary reconstruction without the necessity to resample archaeological material. This is of particular interest for archaeological and palaeontological assemblages with small-sized samples or samples too valuable for repeated destructive analyses (e.g., human remains). Additionally, collagen extraction does not always provide δ^{15}N and δ^{13}C results due to, for example, too low collagen content in the sample. However, if the dissolved phase is collected for Zn isotope analysis, these samples may still provide valuable dietary information and are thus not completely "lost".

3.3 Baseline carbon and nitrogen isotope variability recorded in *P. hispida* bones

High geographic variability in consumer tissue bulk δ^{15}N and δ^{13}C values limits their use as dietary proxies when studying highly mobile species or combining multiple geographically distinct populations. Post-hoc Games-Howell and Tukey pair-wise comparisons demonstrate a large heterogeneity in δ^{15}N and δ^{13}C values between archaeological populations (Supplementary Figure 6-7). Most of the differences can be linked to geographic groups resulting in δ^{15}N and δ^{13}C values from populations of different regions plotting in distinct groups on δ^{15}N *versus* δ^{13}C plots (Figure 1). We grouped sites from the Bering/Chukchi Sea, Amundsen and Coronation Gulf, CAA, North Water Polynya, Hudson Bay, and sites influenced by the

Labrador Sea in the Hudson Strait and Frobisher Bay (Figure 1 a). The most likely reasons for varying mean *P. hispida* bone $\delta^{15}N$ and $\delta^{13}C$ values between the sites is a potential difference in diet between populations, or variations in food web baseline $\delta^{15}N$ and $\delta^{13}C$ values.

A varying degree of high and low trophic level food (from the same basal organic matter source) can impact $\delta^{15}N$ values but would have very little or no effect on $\delta^{13}C$ values. Therefore, feeding on a different trophic level may contribute to $\delta^{15}N$ variability between the archaeological sites, but cannot explain the differences observed in their $\delta^{13}C$ values. Different populations may have also relied to a varying degree on benthic *versus* pelagic foraging. Benthic animals tend to be ^{13}C and ^{15}N enriched compared to pelagic animals[33]. However, a more benthic *versus* pelagic diet alone is an unlikely explanation for the full range of bone $\delta^{15}N$ and $\delta^{13}C$ values observed between the archaeological sites in this study. Seasonal shifts in modern *P. hispida* muscle $\delta^{13}C$ values interpreted as changes in diet from a pelagic open-water to a more ice-cover and/or benthic diet were less than 1 ‰[7]. As with muscle $\delta^{13}C$ values, these authors observed a similar pattern for muscle $\delta^{15}N$ values with lower values during the open water period. However, the shift in $\delta^{15}N$ was even lower and statistically non-significant[7]. Still, $\delta^{15}N$ and $\delta^{13}C$ values have a weak correlation for *P. hispida* (Figure 1 b) among all *P. hispida* samples (R^2=0.21, $p < 0.05$), which could indicate that differences in food source among populations contributes to the observed $\delta^{15}N$ and $\delta^{13}C$ site variability. However, the correlation between $\delta^{15}N$ and $\delta^{13}C$ values could also be explained by baseline variability. Depending on the controlling factor, baseline $\delta^{15}N$ and $\delta^{13}C$ variations may follow the same direction, for example with lower values for both with decreasing productivity and/or increased terrestrial nutrient input[34].

Here we observe similar trends in mean $\delta^{13}C$ values from *P. hispida* bone collagen as observed in modern POM[35] with generally higher values at sites with connections to more open marine areas and lower values in the CAA, Hudson Bay and Beaufort Sea (Figure 1, 2 a, b). Our archaeological data also agrees in variability and general geographic spacing with modern *P. hispida* muscle tissue $\delta^{13}C$ variability observed throughout the Arctic. Modern muscle tissue $\delta^{13}C$ values can vary up to 5 ‰ between geographically distinct populations[4,7,36,37]. As with the archaeological record, the lowest values in modern *P. hispida* tissue are also observed close to the Canadian mainland[2,6]. Despite investigating samples from only one area in the Bering Strait/Southern Chukchi Sea, our results agree well with the documented pronounced west-east ^{13}C depletion in consumers throughout the Chukchi Sea and Beaufort Sea[34,38,39,40]. The lowest mean $\delta^{13}C$ values in *P. hispida* bone collagen were also reported from the area SE Beaufort Sea/Amundsen Gulf extending even into the Coronation Gulf, while the highest mean value can be found in bones from the Bering Strait (Figure 1, 2 a). The up to 3.4 ‰ lower mean $\delta^{13}C$ values between *P. hispida* bone collagen from the SE Beaufort Sea/Amundsen Gulf relative to the Bering Strait sites is comparable to previously reported $\delta^{13}C$ gradients for zooplankton (~4.8 ‰, ~3.3 -3.8 ‰)[34,39], secondary consumers (< 2.4 ‰)[38] and filter feeders (< 6.4 ‰)[38], between these water bodies. These lower $\delta^{13}C$ values in bones of the SE Beaufort Sea/Amundsen Gulf sites are likely related to terrestrial organic matter input from the Mackenzie River[41] lowering baseline $\delta^{13}C$ values.

As for *P. hispida* $\delta^{15}N$ in this study, previous studies have not shown a $\delta^{13}C$ comparable geographic variation of nitrogen isotope compositions between the Bering Sea, Chukchi Sea and Beaufort Sea areas within animals of higher trophic levels[2,38]. We observe the lowest $\delta^{15}N$ values for *P. hispida* bone collagen in the most eastern sites from the Hudson Strait and East Baffin Island in proximity to the Labrador Sea which links the Atlantic to the Hudson Bay and Baffin Bay (Figure 1 a, 2 b). These lower values are in good agreement with zooplankton based $\delta^{15}N$ Atlantic isoscapes, showing an increase in baseline $\delta^{15}N$ values

from the Labrador Sea towards Baffin Bay and CAA values[42]. Corresponding with our archaeological bone collagen $\delta^{15}N$ values (Figure 2 b), modern muscle and liver tissue of *P. hispida* has lower values (14.7 ‰) in populations from the Labrador Sea relative to populations in terrestrial influenced regions close to the Canadian mainland in the CAA (17.2 to 17.9 ‰), such as the Amundsen Gulf and Rae Strait[2,6].

Accordingly, dietary differences between populations may contribute to some of the observed bone $\delta^{15}N$ and $\delta^{13}C$ variability between archaeological sites. However, the carbon and nitrogen isotope variability between the archaeological sites is in good agreement with modern geographical variations from zooplankton, food web and *P. hispida* tissue isotope datasets. We therefore assume geographically varying food web baseline isotope values to be the main factor controlling the major isotopic variability among the sites.

3.4 Trophic level assessment

In order to establish the relationship between bone $\delta^{66}Zn$ and trophic level for the Arctic mammals of this study, we first had to assess the trophic level (TL) of every single animal. As we analyse archaeological material herein, the only other TL indicator available is bulk collagen $\delta^{15}N$. As $\delta^{15}N$ varies due to baseline effects among sites and we do not know the concentrations of Zn ingested nor the dietary $\delta^{66}Zn$ composition, we cannot quantify the true $\delta^{66}Zn$ TL discrimination factors herein. However, by comparing $\delta^{66}Zn$ values of all taxa analysed with their TL (calculated based on bulk collagen $\delta^{15}N$ values) we can estimate $\delta^{66}Zn$ TL discrimination factors. To do so, we used the following equation based on $\delta^{15}N$ values established by Hobson and Welch[43]:

$$\mathrm{TL} = 1 + (\delta^{15}N - 5.4)/3.8 \quad \text{(Supplementary Equation 1)}$$

Where TL is the consumer trophic level and the $\delta^{15}N$ enrichment value is +3.8 ‰ corresponding to the trophic level spacing of TL = +1 between *P. hispida* and *U. maritimus*[43]. In the Lancaster Sound region (LSR) where this relationship was established, *U. maritimus* almost exclusively feed on this specific species of seals[43].

The average trophic level of the Arctic animals for which both $\delta^{15}N$ and $\delta^{66}Zn$ values are available (ref.[18] and this study) calculated following Supplementary Equation 1 are given Supplementary Table 1 (extreme outlier values were excluded; see also Supplementary Figure 8)

The $\delta^{15}N$ trophic levels are in good agreement with the previous findings of Hobson and Welch[43] for the LSR as well as other locations[36,44,45]. However, these TL estimations only represent oversimplified estimations, not considering population specific dietary differences, location specific baseline variations and organism specific trophic and tissue-type enrichment factors.

Based on the nitrogen isotope data we established two equations to estimate the trophic level of marine mammals using bone $\delta^{66}Zn$ values (Supplementary Equations 2 and 3).

$$\mathrm{TL} = -2.76 * \delta^{66}Zn + 5.48 \quad \text{(Supplementary Equation 2)}$$

With a R^2=0.57.

Without including the *O. rosmarus* bones, the relationship becomes:

$$\mathrm{TL} = -2.64 * \delta^{66}Zn + 5.48 \quad \text{(Supplementary Equation 3)}$$

With a R^2=0.64.

Supplementary Equation 3 predicts a δ^{66}Zn bone value of 1.32 ‰ for a TL = 2, and 0.94 ‰ for a TL = 3 which is very close to that seen in bones of terrestrial herbivores (TL = 2) and carnivores (TL = 3)[13]. Applying Supplementary Equation 3 to modern terrestrial mammal bones from Koobi Fora (Kenya)[13] gives us a TL of 1.8 for the average herbivore values (*Madoqua guentheri, Tragelaphus imberis, Litocranius walleri, Damaliscus korrigum, Oryx beisa, Equus burchelli*) and 3.3 for combined carnivores of that study (*Felis leo, Caracal caracal, Canis sp., Felis silvestris*).

The δ^{66}Zn TL estimates are generally in agreement with the species respective trophic levels (Supplementary Table 2, Supplementary Figure 8). *Erignathus barbatus* δ^{66}Zn estimated TL is lower than reported by Hobson & Welch[43] and Hobson et al.[36] (TL = 4.0 to 4.3), but it is close to the TL estimate (TL = 3.4) of Pauly et al.[44]. *Odobenus rosmarus* bone δ^{66}Zn values do not seem to reflect their trophic level relative to the other taxa analysed. δ^{66}Zn TL estimates for *O. rosmarus* place it at 4.0 to 4.1, which is too high based on its diet. *Odobenus rosmarus* feed mostly on molluscs, especially filter-feeding bivalves such as *Mya truncata* and *Hiatella arctica*[46]. *Odobenus rosmarus* is therefore primarily a benthic feeder, whereas the other mammals primarily feed along a pelagic-based trophic chain (pelagic POM - zooplankton - planktivorous fish - piscivorous fish - piscivorous mammals - carnivorous mammals). *Odobenus rosmarus* might be considered as feeding of a different food web, we thus recommend the use of Supplementary Equation 3 for calculating mammal TL based on bone δ^{66}Zn values. Benthic food webs can also differ in δ^{15}N and δ^{13}C compared to pelagic food webs[33,47]. Additional δ^{66}Zn analysis is required to investigate whether a primarily benthic invertebrate based diet results in a different δ^{66}Zn baseline or different Zn fractionation within consumers relative to consumers feeding along a primarily pelagic-based trophic chain. If so, then combining δ^{66}Zn with δ^{15}N and δ^{13}C analysis may be a powerful approach to identify not only relative trophic levels, but also habitat use and benthic *versus* pelagic dietary preferences.

Supplementary Figures

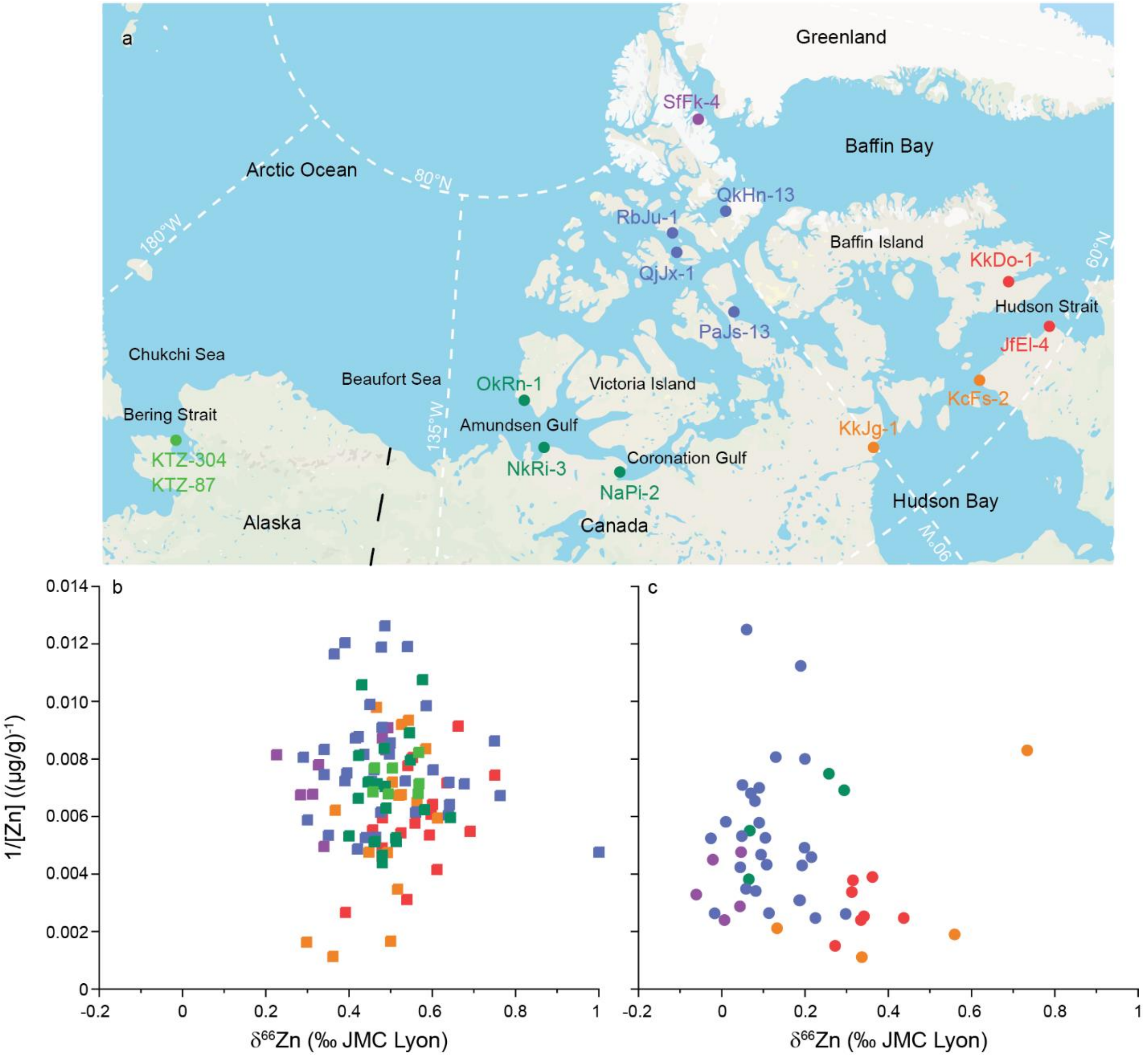

Supplementary Figure 1: δ^{66}Zn versus zinc concentrations [Zn] expressed as 1/[Zn] for n=105 *P. hispida* (b) and n=47 *U. maritimus* (c) bone samples. Samples are colour coded after map (a) indicating the archaeological sites analysed. Colour coding: Light green for the Bering Strait; dark green for the Amundsen and Coronation Gulf; blue for the CAA; orange for the Hudson Bay; purple for North Water Polynya; and red for sites influenced by the Labrador Sea in the Hudson Strait and Frobisher Bay. An extreme outlier *P. hispida* value (δ^{66}Zn = 1.00 ‰, from QjJx-1[18]) is included. The schematic map was redrawn and modified using Adobe Illustrator CS6 after www.google.com/maps.

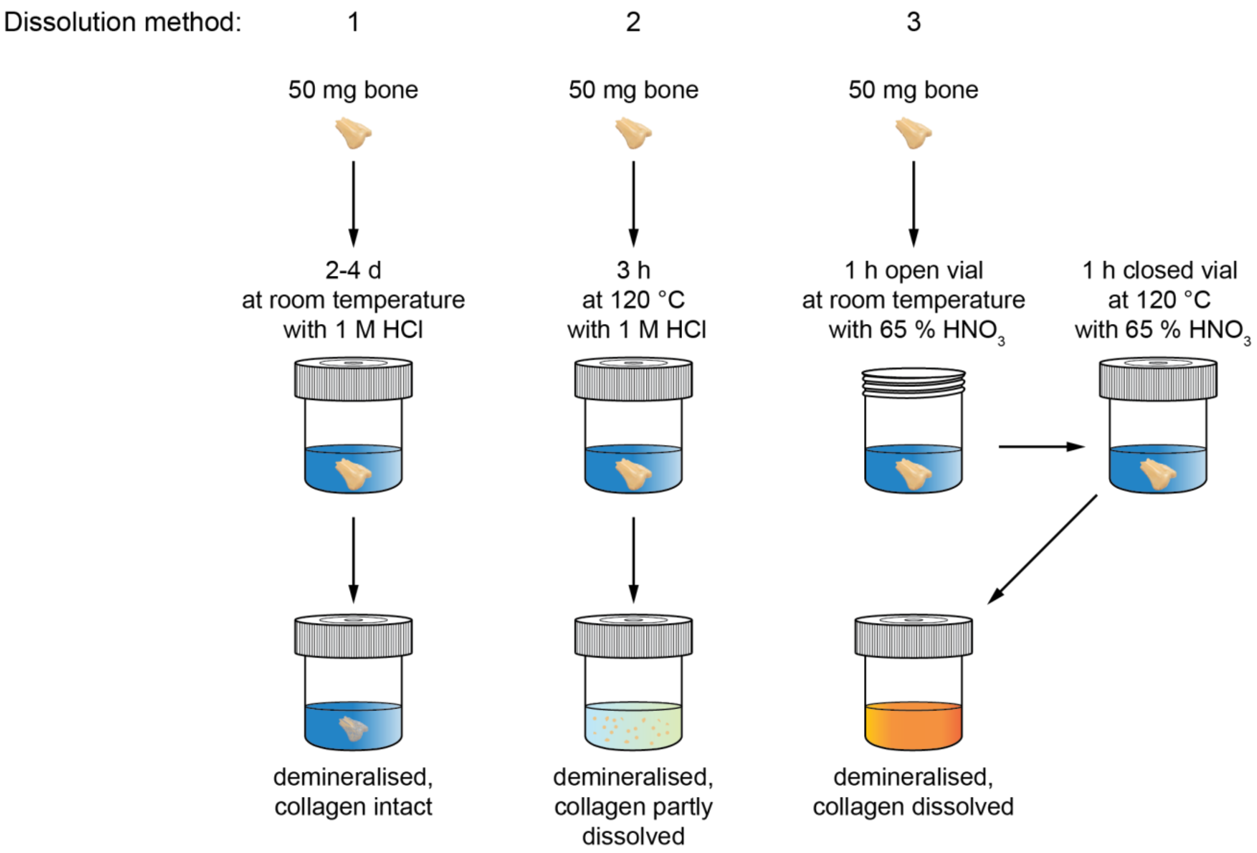

Supplementary Figure 2: Dissolution methods used to test the impact of collagen on bone mineral $\delta^{66}Zn$ values.

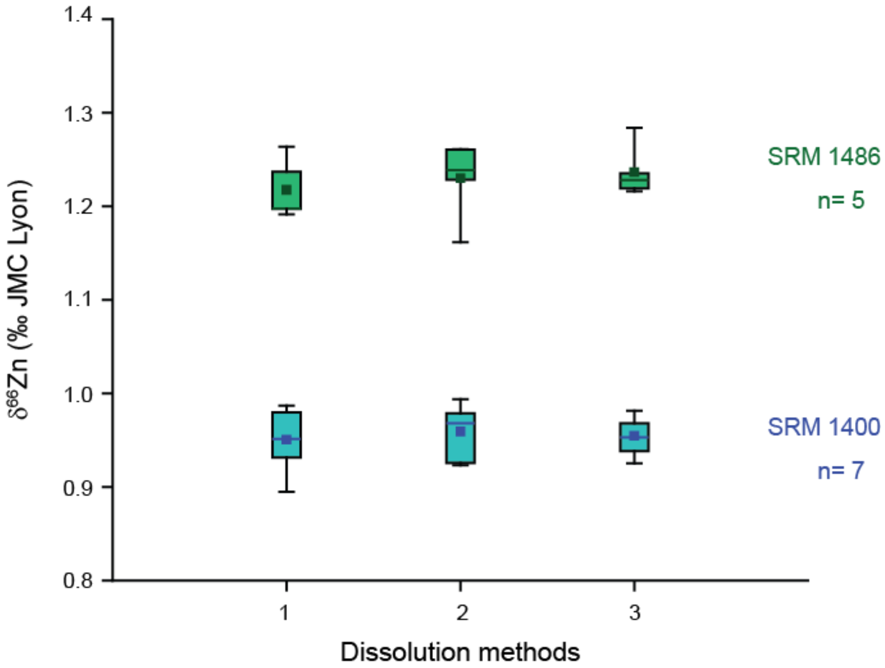

Supplementary Figure 3: Bone ash NIST SRM 1400 and bone meal NIST SRM 1486 $\delta^{66}Zn$ values obtained by applying different dissolution methods described in Supplementary Methods 2.1 and Supplementary Figure 2. The boxes from the box and whisker plots represent the 25th–75th percentiles, with the median as a bold horizontal line and mean value as a dark filled box; n indicates the number of SRM standards measured for each dissolution method.

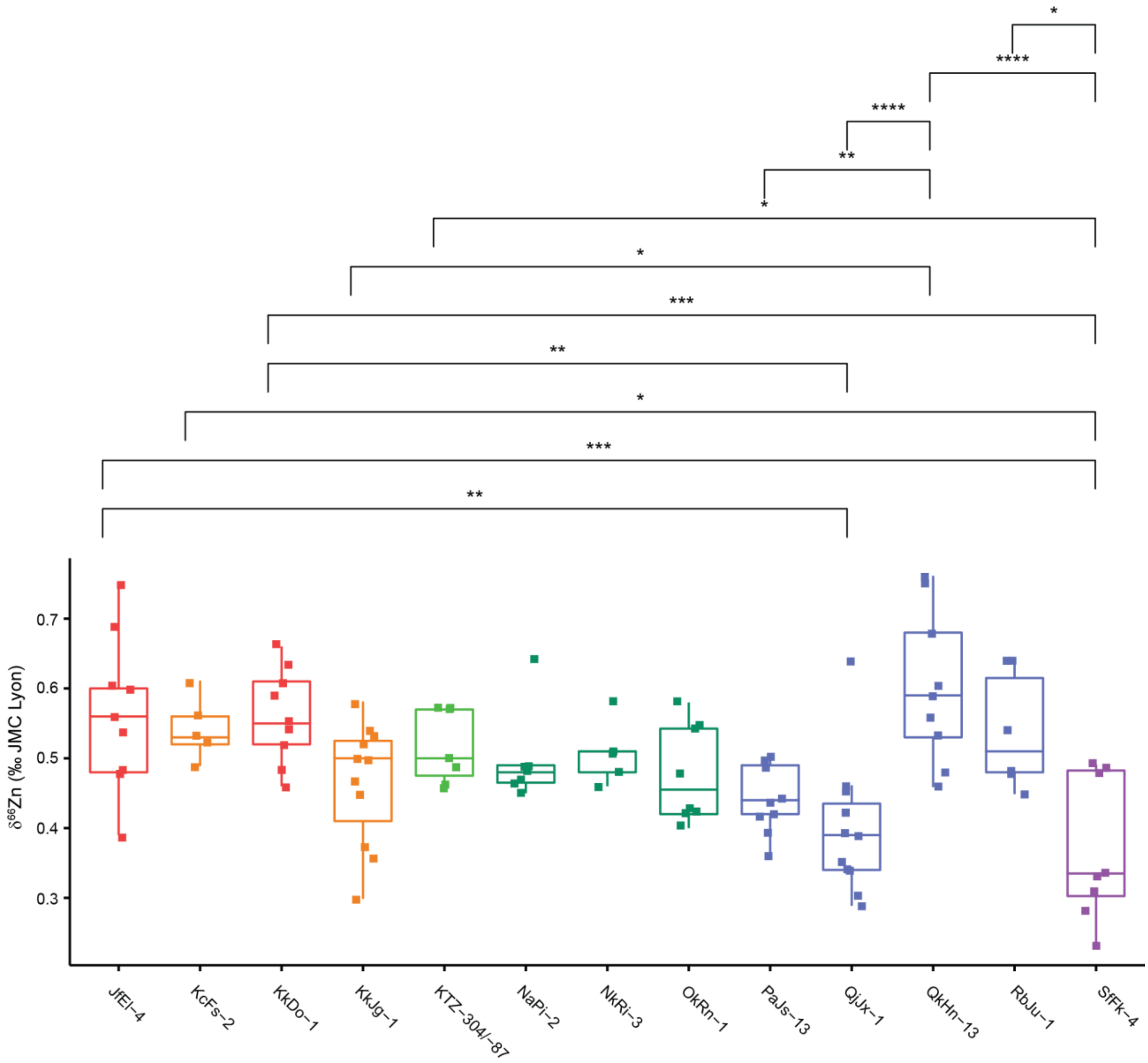

Supplementary Figure 4: Results for post-hoc Tukey pair-wise comparisons between sites (indicated by their Borden code) for $\delta^{66}Zn$ values of *P. hispida* bones (n=104). The boxes from the box and whisker plots represent the 25th–75th percentiles, with the median as a bold horizontal line. Significance level is indicated by "*" (p-value < 0.05), "**" (p-value < 0.005), "***" (p-value < 0.0005) and "****" (p-value < 0.00005).

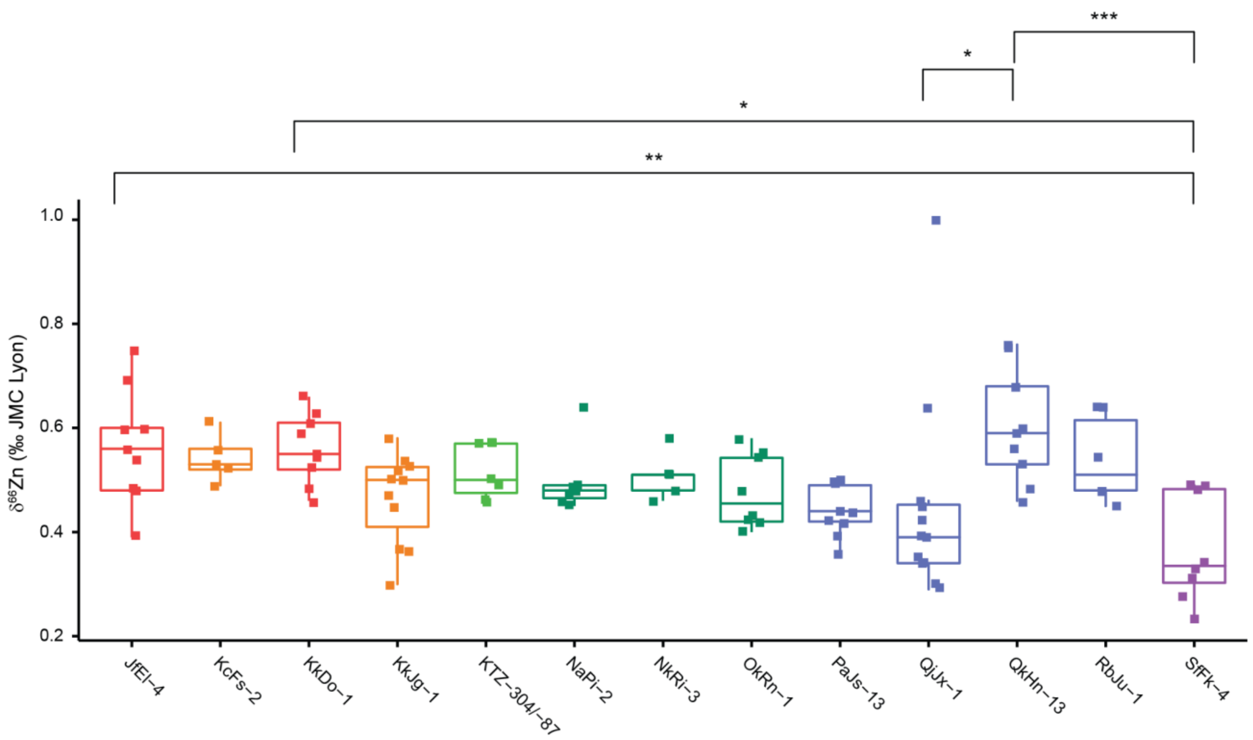

Supplementary Figure 5: Results for post-hoc Tukey pair-wise comparisons between sites (indicated by their Borden code) for $\delta^{66}Zn$ values of *P. hispida* bones including an extreme outlier value from Little Cornwallis Island[15] (n=105). The boxes from the box and whisker plots represent the 25th–75th percentiles, with the median as a bold horizontal line. Significance level is indicated by "*" (*p-value* < 0.05), "**" (*p-value* < 0.005), "***" (*p-value* < 0.0005) and "****" (*p-value* < 0.00005).

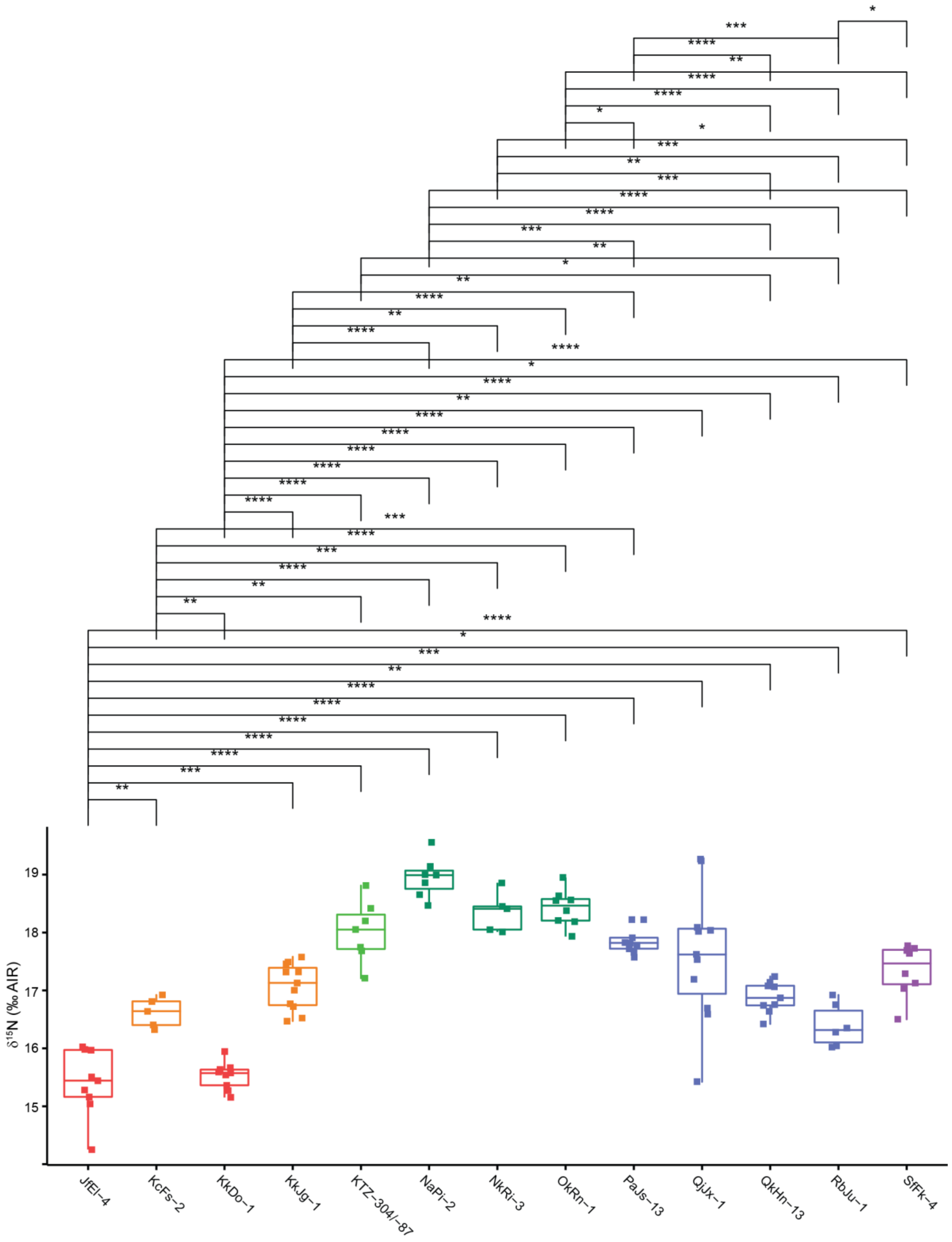
δ15N (‰ AIR)
19
18
17
16
15
JfEl−4
KcFs−2
KkDo−1
KkJg−1
KTZ−304/−87
NaPi−2
NkRi−3
OkRn−1
PaJs−13
QjJx−1
QkHn−13
RbJu−1
SfFk−4

Supplementary Figure 6: Results for post-hoc Games-Howell pair-wise comparisons between sites (indicated by their Borden code) for $\delta^{15}N$ values of *P. hispida* bones (n=104). The boxes from the box and whisker plots represent the 25th–75th percentiles, with the median as a bold horizontal line. Significance level is indicated by "*" (*p-value* < 0.05), "**" (*p-value* < 0.005), "***" (*p-value* < 0.0005) and "****" (*p-value* < 0.00005).

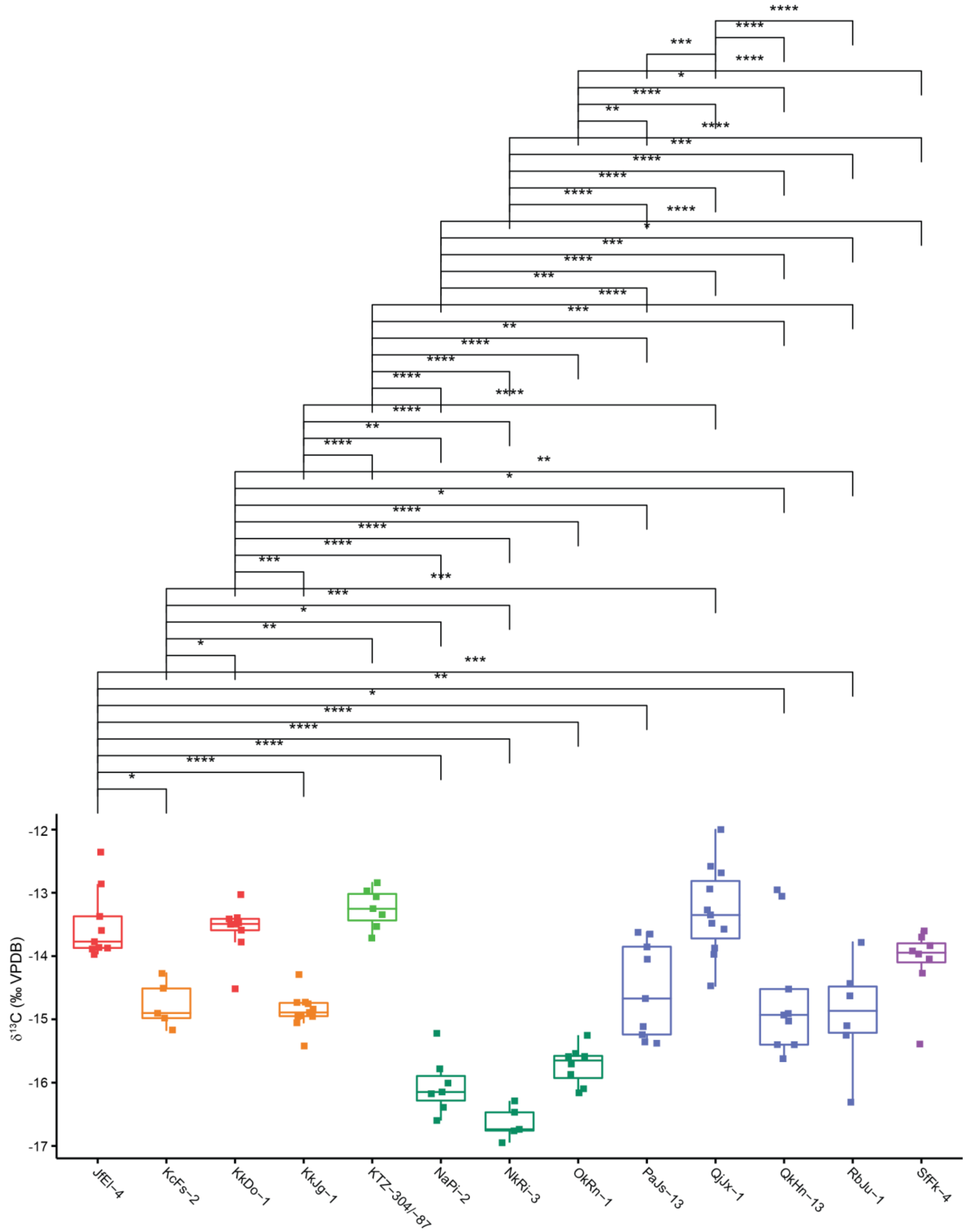

Supplementary Figure 7: Results for post-hoc Tukey pair-wise comparisons between sites (indicated by their Borden code) for $\delta^{13}C$ values of *P. hispida* bones (n=104). The boxes from the box and whisker plots

represent the 25th–75th percentiles, with the median as a bold horizontal line. Significance level is indicated by "*" (*p-value* < 0.05), "**" (*p-value* < 0.005), "***" (*p-value* < 0.0005) and "****" (*p-value* < 0.00005).

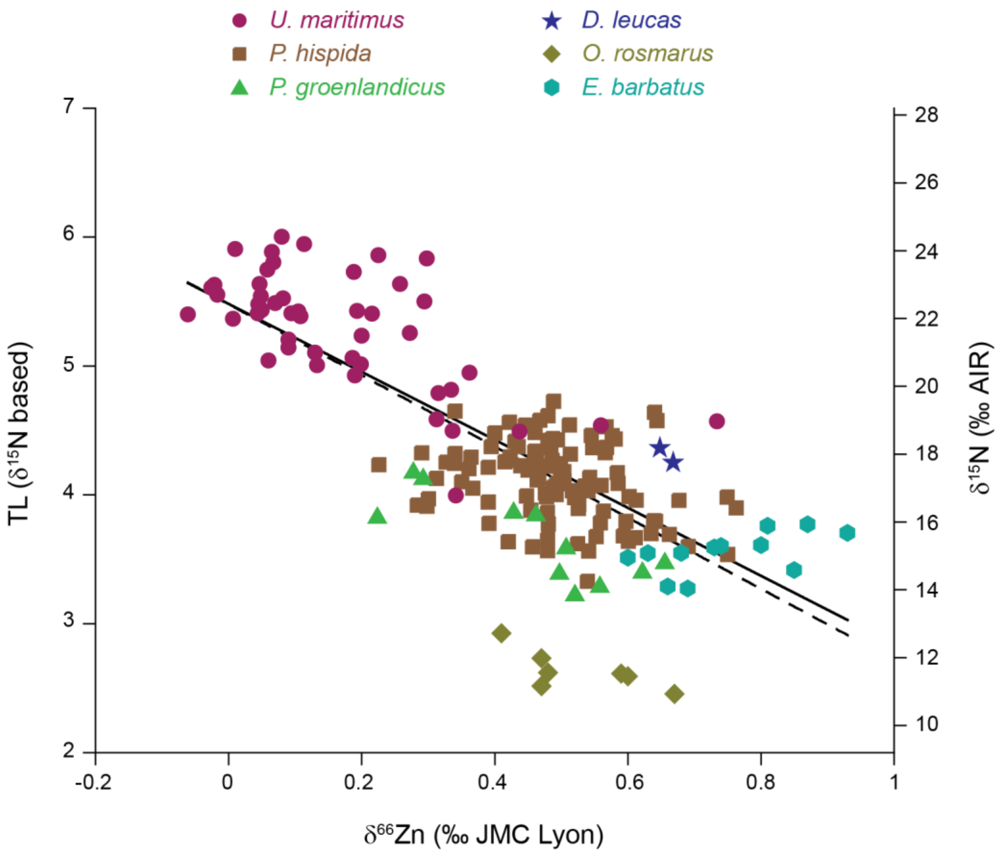

Supplementary Figure 8: TL calculated using $\delta^{15}N$ and $\delta^{15}N$ *versus* $\delta^{66}Zn$ bone values for all species analysed herein together with ref.[18]. Dashed line represents the linear fit including *O. rosmarus* samples (p-value < 0.05; R^2 = 0.57; n = 183, Supplementary Equation 2), solid line represents the linear fit excluding *O. rosmarus* samples (p-value < 0.05; R^2 = 0.64; n = 176, Supplementary Equation 3). Two extreme outlier samples based on their $\delta^{66}Zn$ values from the QjJx-1 site (*P. hispida* 1.00 ‰; *E. barbatus* 1.39 ‰)[18] and one based on its $\delta^{15}N$ value from the JfEl-4 site (*D. leucas*? 11.82 ‰) were excluded. Additionally, only cortical bone $\delta^{66}Zn$ values for *O. rosmarus* from the QjJx-1 site were used.

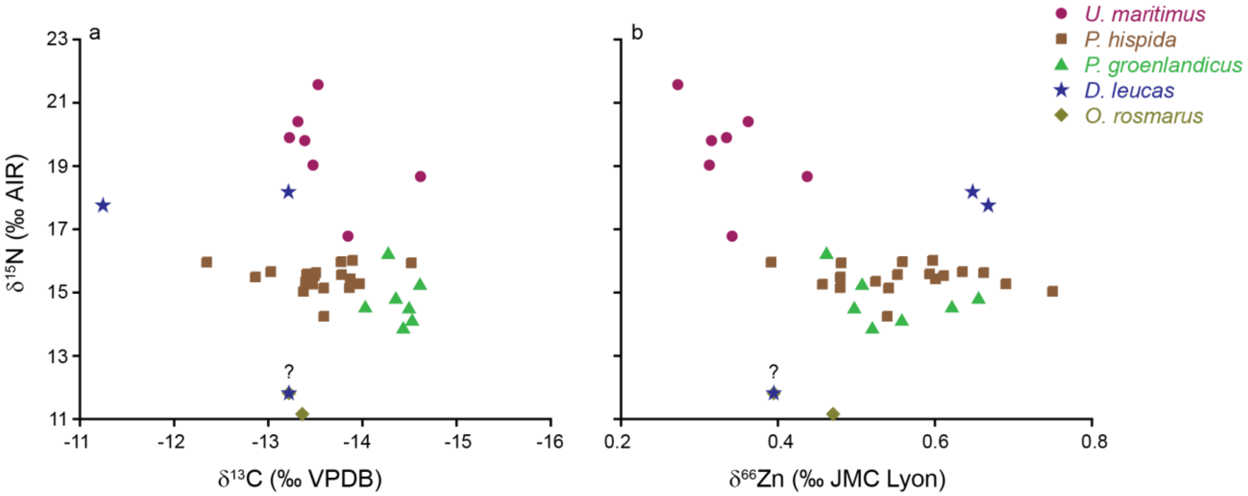

Supplementary Figure 9: $\delta^{15}N$ *versus* $\delta^{13}C$ (a), $\delta^{15}N$ *versus* $\delta^{66}Zn$ (b) of *U. maritimus* (dots, n=7), *P. hispida* (squares, n=18), *P. groenlandicus* (triangle, n=7), *D. leucas* (star, n=3) and *O. rosmarus* (diamond, n=1) bones for the combined KkDo-1 and JfEl-4 sites. A bone sample identified as a *D. leucas* with an unusually low $\delta^{15}N$ value is indicated by a star overlying a diamond and marked with a question mark. We cannot exclude the possibility of taxonomic misidentification for that sample.

Supplementary Tables

Supplementary Table 1. *$\delta^{15}N$ based trophic level (TL) estimates following Supplementary Equation 1[43]. SD = standard deviation, n = number of individuals/bone samples. Two extreme outlier samples based on their $\delta^{66}Zn$ values from the QjJx-1 site (P. hispida 1.00 ‰; E. barbatus 1.39 ‰)[18] and one based on its $\delta^{15}N$ value from the JfEl-4 site (D. leucas? 11.82 ‰) were excluded. Additionally, only cortical bone $\delta^{66}Zn$ values for O. rosmarus from the QjJx-1 site were used.*

	This study and Jaouen et al., 2016[18]			Hobson & Welch (1992)[43]	
**Species**	**$\delta^{15}N$ TL**	**SD**	**n**	**$\delta^{15}N$ TL**	**n**
U. maritimus	5.3	0.45	47	5.1	3
P. hispida	4.1	0.30	104	4.1	9
D. leucas	4.3	0.05	2	3.9	6
P. groenlandicus	3.7	0.32	11		
E. barbatus	3.6	0.16	12	4.0	4
O. rosmarus	2.6	0.14	7	2.9	6

Supplementary Table 2. *$\delta^{66}Zn$ based trophic level (TL) estimates following Supplementary Equation 2 and 3. SD = standard deviation, n = number of individuals/bone samples. Two extreme outlier samples based on their $\delta^{66}Zn$ values from the QjJx-1 site (P. hispida 1.00 ‰; E. barbatus 1.39 ‰)[18] and one based on its $\delta^{15}N$ value from the JfEl-4 site (D. leucas? 11.82 ‰) were excluded. Additionally, only cortical bone $\delta^{66}Zn$ values for O. rosmarus from the QjJx-1 site were used.*

	Supplementary Equation 2		Supplementary Equation 3		
**Species**	**$\delta^{66}Zn$ TL**	**SD**	**$\delta^{66}Zn$ TL**	**SD**	**n**
U. maritimus	5.01	0.44	5.03	0.42	47
P. hispida	4.12	0.29	4.18	0.27	104
D. leucas	3.66	0.04	3.74	0.04	2
P. groenlandicus	4.21	0.39	4.27	0.37	11
E. barbatus	3.28	0.56	3.37	0.54	12
O. rosmarus	4.02	0.26	4.09	0.25	7

Supplementary Table 3. *Archaeological sites analysed in this study with approximate age of the bone samples and additional references.*

Site	Approximate age (calibrated years BP)	Reference
KTZ-304	650–850	Darwent *et al.* (2013)[48]
KTZ-087	350–550	Darwent *et al.* (2013)[48]
NkRi-3	650–750	Moody & Hodgetts (2013)[28]
NaPi-2	550–650	Morrison (1983)[49]
RbJu-1	3900–4100	McGhee (1979)[50]
QkHn-13	3400–3800	Helmer (1991)[51]
OkRn-1	300–500	Kotar (2016)[52]
OlRr-1	650-750	Manning (1956)[53]
QjJx-1	600–1100	Rick (1980)[54]
PaJs-13	550–650	Savelle & Habu (2004)[55]
PcJq-5	550–650	Rick (1980)[54]
PeJr-1	550–650	Rick (1980)[54]
KkJg-1	325–500	Staab (1979)[56]<br>Dyke *et al.* (2019)[57]
KcFs-2	450–1450	Thompson (2011)[58]
JfEl-4	550–700	Badgley (1980)[59]
KkDo-1	650–150	Stenton (1987)[60]
SfFk-4	550–650	Howse (2013)[61]

Supplementary Table 4. *Standard deviations for the carbon and nitrogen isotopic compositions of the calibration standards used in all analytical sessions associated with the data presented in this paper.*

Standard	*n*	δ^{13}C (±1σ)	δ^{15}N (±1σ)
USGS40	450	0.06	0.18
USGS41	438	0.18	0.12

Supplementary Table 5. *Isotopic reference materials used to monitor internal accuracy and precision.*

Standard	Material	Mean δ^{13}C (‰, VPDB)	Mean δ^{15}N (‰, AIR)
MET	Methionine[a]	−28.61±0.10	−5.04±0.13
NIST-1577c	Bovine liver[a]	−17.52±0.09	+8.15±0.14
SRM-1	Caribou bone collagen[a]	−19.40±0.08	+1.83±0.11
SRM-2	Walrus bone collagen[a]	−14.77±0.12	+15.59±0.13
USGS42	Human hair	−21.09±0.10	+8.05±0.10
USGS43	Human hair	−21.28±0.10	+8.44±0.10
IAEA-CH-3	Cellulose	−24.72±0.04	–

a. Internal standard with mean isotopic compositions representing long-term values as measured in three different laboratories.

Supplementary Table 6. *Mean and standard deviations of all the check (QA) standards analysed in the analytical sessions associated with data presented in this paper.*

		$\delta^{13}C$ (‰, VPDB)			$\delta^{15}N$ (‰, AIR)		
**Standard**	***n***	**Mean**	**±**	**1σ**	**Mean**	**±**	**1σ**
MET	357	−28.61	±	0.07	−5.03	±	0.13
NIST-1577c	134	−17.52	±	0.09	+8.15	±	0.12
SRM-1	123	−19.32	±	0.07	+1.81	±	0.12
SRM-2	114	−14.74	±	0.10	+15.60	±	0.08
USGS42	4	−21.09	±	0.02	+7.98	±	0.03
USGS43	3	−21.28	±	0.02	+8.41	±	0.06
IAEA-CH-3	4	−24.70	±	0.05			

Supplementary References

1. Reeves, R. R. *Distribution, abundance and biology of ringed seals (Phoca hispida): an overview.* In: *Ringed Seals in the North Atlantic*, (eds Heide-Jørgensen, M. P. & Lydersen, C.) 9-45, (NAMMCO Scientific Publications, 1998).
2. Dehn, L. A. et al. Feeding ecology of phocid seals and some walrus in the Alaskan and Canadian Arctic as determined by stomach contents and stable isotope analysis. *Polar Biol.* **30,** 167-181 (2007).
3. Lowry, L. F., Frost, K. J. & Burns, J. J. Variability in the diet of ringed seals, *Phoca hispida*, in Alaska. *Can. J. Fish. Aquat. Sci.* **37,** 2254-2261 (1980).
4. Matley, J. K., Fisk, A. T. & Dick, T. A. Foraging ecology of ringed seals (*Pusa hispida*), beluga whales (*Delphinapterus leucas*) and narwhals (*Monodon monoceros*) in the Canadian High Arctic determined by stomach content and stable isotope analysis. *Polar Res.* **34,** 24295 (2015).
5. Born, E. W., Teilmann, J., Acquarone, M. & Riget, F. F. Habitat use of ringed seals (*Phoca hispida*) in the North Water area (North Baffin Bay). *Arctic* **57,** 129-142 (2004).
6. Butt, C. M., Mabury, S. A., Kwan, M., Wang, X. & Muir, D. C. Spatial trends of perfluoroalkyl compounds in ringed seals (*Phoca hispida*) from the Canadian Arctic. *Environ. Toxicol. Chem.* **27**, 542-553 (2008).
7. Young, B. G. & Ferguson, S. H. Seasons of the ringed seal: pelagic open-water hyperphagy, benthic feeding over winter and spring fasting during molt. *Wildl. Res.* **40,** 52-60 (2013).
8. Lunn, N. J. et al. *Polar bear management in Canada 1997–2000*. In: *Proceedings of the 13th Working Meeting of the IUCN/SSC Polar Bear Specialist Group, 23–28 June 2001, Nuuk, Greenland. Occasional Paper 26,* (eds Lunn, N. J., Schliebe, S. & Born, E. W.), 41-52, (IUCN, 2002).
9. Iverson, S. J., Stirling, I. & Lang, S. L. C. *Spatial and temporal variation in the diets of polar bears across the Canadian Arctic: indicators of changes in prey populations and*

environment. In: *Top predators in marine ecosystems*, (eds Boyd, I. L., Wanless, S. & Camphuysen, C. J.), 98–117, (Cambridge University Press, 2006).
10. Thiemann, G. W., Iverson, S. J. & Stirling, I. Polar bear diets and arctic marine food webs: insights from fatty acid analysis. *Ecol. Monogr.*, **78,** 591-613 (2008).
11. Laidre, K. L., Stirling, I., Estes, J. A., Kochnev, A. & Roberts, J. Historical and potential future importance of large whales as food for polar bears. *Front. Ecol. Environ.* **16,** 515-524 (2018).
12. Ramsay, M. A. & Hobson, K. A. Polar bears make little use of terrestrial food webs: evidence from stable-carbon isotope analysis. *Oecologia* **86,** 598-600 (1991).
13. Jaouen, K., Beasley, M., Schoeninger, M., Hublin, J. J. & Richards, M. P. Zinc isotope ratios of bones and teeth as new dietary indicators: results from a modern food web (Koobi Fora, Kenya). *Sci. Rep.* **6,** 26281 (2016).
14. Bourgon, N. et al. Zinc isotopes in Late Pleistocene fossil teeth from a Southeast Asian cave setting preserve paleodietary information. *PNAS* **117,** 4675-4681 (2020).
15. Qi H., Coplen T. B., Geilmann H., Brand W. A. & Böhlke J. K. Two new organic reference materials for δ^{13}C and δ^{15}N measurements and a new value for the δ^{13}C of NBS 22 oil. *Rapid Commun. Mass Spectrom.* **17,** 2483-2487 (2003).
16. Szpak P., Metcalfe J. Z. & Macdonald R. A. Best Practices for Calibrating and Reporting Stable Isotope Measurements in Archaeology. *J. Archaeol. Sci. Rep.* **13,** 609-616 (2017).
17. Magnusson, B., Näykki, T., Hovind, H. & Krysell, M. *Handbook for the calculation of measurement uncertainty in environmental laboratories.* (Nordtest Technical Report 537, 2012).
18. Jaouen, K., Szpak, P. & Richards, M. P. Zinc Isotope Ratios as Indicators of Diet and Trophic Level in Arctic Marine Mammals. *PLoS One* **11,** e0152299 (2016).
19. DeNiro M.J. Postmortem preservation and alteration of in vivo bone collagen isotope ratios in relation to palaeodietary reconstruction. *Nature* **317**, 806-809 (1985).
20. Ambrose, S. H. Preparation and characterization of bone and tooth collagen for isotopic analysis. *J. Archaeol. Sci.* **17,** 431-451 (1990).
21. Hedges, R. E. Bone diagenesis: an overview of processes. *Archaeometry* **44,** 319-328 (2002).
22. Trueman, C. N., Behrensmeyer, A. K., Tuross, N. & Weiner, S. Mineralogical and compositional changes in bones exposed on soil surfaces in Amboseli National Park, Kenya: diagenetic mechanisms and the role of sediment pore fluids. *J. Archaeol. Sci.* **31,** 721-739 (2004).
23. Reynard, B. & Balter, V. Trace elements and their isotopes in bones and teeth: Diet, environments, diagenesis, and dating of archeological and paleontological samples. *Palaeogeogr. Palaeoclimatol. Palaeoecol.* **416,** 4-16 (2014).
24. Sieber, M. et al. Cycling of zinc and its isotopes across multiple zones of the Southern Ocean: Insights from the Antarctic Circumnavigation Expedition. *Geochim. Cosmochim. Acta* **268**, 310-324 (2020).

25. Vance, D., de Souza, G. F., Zhao, Y., Cullen, J. T. & Lohan, M. C. The relationship between zinc, its isotopes, and the major nutrients in the North-East Pacific. *Earth Planet. Sci. Lett.* **525,** 115748 (2019).
26. Twining, B. S., Rauschenberg, S., Morton, P. L. & Vogt, S. Metal contents of phytoplankton and labile particulate material in the North Atlantic Ocean. *Prog. Oceanogr.* **137,** 261-283 (2015).
27. Jensen, L. T. et al. Biogeochemical cycling of dissolved zinc in the Western Arctic (Arctic GEOTRACES GN01). *Global Biogeochem. Cycles* **33,** 343-369 (2019).
28. Moody, J. F. & Hodgetts, L. M. Subsistence practices of pioneering Thule–Inuit: A faunal analysis of Tiktalik. *Arctic Anthropol.* **50,** 4-24 (2013).
29. Lone, K., Hamilton, C. D., Aars, J., Lydersen, C. & Kovacs, K. M. Summer habitat selection by ringed seals (*Pusa hispida*) in the drifting sea ice of the northern Barents Sea. *Polar Res.* **38,** 3483 (2019).
30. Tang, Y., Chappell, H. F., Dove, M. T., Reeder, R. J. & Lee, Y. J. Zinc incorporation into hydroxylapatite. *Biomaterials* **30,** 2864-2872 (2009).
31. Mayer, I., Apfelbaum, F. & Featherstone, J. D. B. Zinc ions in synthetic carbonated hydroxyapatites. *Arch. Oral Biol.* **39,** 87-90 (1994).
32. Murray, E. J. & Messer, H. H. Turnover of bone zinc during normal and accelerated bone loss in rats. *J. Nutr.* **111,** 1641-1647 (1981).
33. Iken, K., Bluhm, B. A. & Gradinger, R. Food web structure in the high Arctic Canada Basin: evidence from $\delta^{13}C$ and $\delta^{15}N$ analysis. *Polar Biol.* **28,** 238-249 (2005).
34. Schell, D. M., Barnett, B. A. & Vinette, K. A. Carbon and nitrogen isotope ratios in zooplankton of the Bering, Chukchi and Beaufort seas. *Mar. Ecol. Prog. Ser.* **162,** 11-23 (1998).
35. De la Vega, C., Jeffreys, R. M., Tuerena, R., Ganeshram, R. & Mahaffey, C. Temporal and spatial trends in marine carbon isotopes in the Arctic Ocean and implications for food web studies. *Glob. Chang. Biol.* **25,** 4116-4130 (2019).
36. Hobson, K. A. et al. A stable isotope ($\delta^{13}C$, $\delta^{15}N$) model for the North Water food web: implications for evaluating trophodynamics and the flow of energy and contaminants. *Deep Sea Res. Part II Top. Stud. Oceanogr.* **49,** 5131-5150 (2002).
37. Muir, D. C. G. et al. Can seal eating explain elevated levels of PCBs and organochlorine pesticides in walrus blubber from eastern Hudson Bay (Canada)?. *Environ. Pollut.* **90,** 335-348 (1995).
38. Dunton, K. H., Saupe, S. M., Golikov, A. N., Schell, D. M. & Schonberg, S. V. Trophic relationships and isotopic gradients among arctic and subarctic marine fauna. *Mar. Ecol. Prog. Ser.* **56,** 89-97 (1989).
39. Saupe, S. M., Schell, D. M. & Griffiths, W. B. Carbon-isotope ratio gradients in western arctic zooplankton. *Mar. Biol.* **103,** 427-432 (1989).

40. Pomerleau, C. et al. Pan-Arctic concentrations of mercury and stable isotope ratios of carbon ($\delta^{13}C$) and nitrogen ($\delta^{15}N$) in marine zooplankton. *Sci. Total Environ.* **551,** 92-100 (2016).
41. Parsons, T. R. et al. Autotrophic and heterotrophic production in the Mackenzie River/Beaufort Sea estuary. *Polar Biol.* **9,** 261-266 (1989).
42. McMahon, K. W., Hamady, L. L. & Thorrold, S. R. A review of ecogeochemistry approaches to estimating movements of marine animals. *Limnol. Oceanogr.* **58,** 697-714 (2013).
43. Hobson, K. A. & Welch, H. E. Determination of trophic relationships within a high Arctic marine food web using $\delta^{13}C$ and $\delta^{15}N$ analysis. *Mar. Ecol. Prog. Ser.* **84,** 9-18 (1992).
44. Pauly, D., Trites, A. W., Capuli, E. & Christensen, V. Diet composition and trophic levels of marine mammals. *ICES J. Mar. Sci.* **55,** 467-481 (1998).
45. Haug, T. et al. Trophic level and fatty acids in harp seals compared with common minke whales in the Barents Sea. *Mar. Biol. Res.* **13,** 919-932 (2017).
46. Fisher, K. I. & Stewart, R. E. A. Summer foods of Atlantic walrus, *Odobenus rosmarus rosmarus*, in northern Foxe Basin, Northwest Territories. *Can. J. Zool.* **75,** 1166-1175 (1997).
47. France, R. L. Carbon-13 enrichment in benthic compared to planktonic algae: foodweb implications. *Mar. Ecol. Prog. Ser.* **124,** 307-312 (1995).
48. Darwent, J., Mason, O. K., Hoffecker, J. F. & Darwent, C. M. 1,000 years of house change at Cape Epsenberg, Alaska: A case study in horizontal stratigraphy. *Am. Antiq.* **78,** 433-455 (2013).
49. Morrison, D. A. Thule Sea Mammal Hunting in the Western Central Arctic. *Arctic Anthropol.* **20,** 61-78 (1983).
50. McGhee, R. *The Palaeoeskimo Occupations at Port Refuge, High Arctic Canada. Archaeology Survey of Canada Paper 92.* (Ottawa: National Museums of Canada, 1979).
51. Helmer, J. W. The Palaeo-Eskimo Prehistory of the North Devon Lowlands. *Arctic* **44,** 301-317 (1991).
52. Kotar K. *Variability in Thule Inuit Subsistence Economy: A Faunal Analysis of OkRn-1, Banks Island, N.W.T.* [M.A. Thesis]. (London, Ontario: The University of Western Ontario, 2016).
53. Manning T. H. Narrative of a Second Defence Research Board Expedition to Banks Island, with Notes on the Country and Its History. *Arctic* **9,** 3-77 (1956).
54. Rick, A. M. Non-Cetacean Vertebrate Remains from Two Thule Winter Houses on Somerset Island, N.W.T. *Can. J. Archaeol.* **4,** 99-117 (1980).
55. Savelle, J. M. & Habu, J. A Processual Investigation of a Thule Whale Bone House, Somerset Island, Arctic Canada. *Arctic Anthropol.* **41,** 204-221 (2004).
56. Staab, M. L. *Analysis of faunal material recovered from a Thule Eskimo site on the Island of Silumiut, N.W.T., Canada*. In: *Thule Eskimo Culture: An Anthropological Retrospective.*

Archaeological Survey of Canada Paper No. 88, (Ed. McCartney A. P.), 349-379 (Ottawa: National Museums of Canada, 1979).

57. Dyke, A. S. et al. An assessment of marine reservoir corrections for radiocarbon dates on walrus from the Foxe Basin region of Arctic Canada. *Radiocarbon*, **61,** 67-81 (2019).
58. Thompson, A. K. *A Zooarchaeological Analysis of a Late Dorset Faunal Assemblage from the KcFs-2 Site (Nunavik, Quebec).* [M.A. Thesis]. (Montréal, QC: Université de Montréal, 2011).
59. Badgley, I. Stratigraphy and Habitation Features at DIA. 4 (JfE 1-4), a Dorset Site in Arctic Quebec. *Arctic* **33,** 569-584 (1980).
60. Stenton, D. R. Recent Archaeological Investigations in Frobisher Bay, Baffin Island, N.W.T. *Can. J. Archaeol.* **11,** 13-48 (1987).
61. Howse, L. Revisiting an Early Thule Inuit occupation of Skraeling Island, Canadian High Arctic. *Etud Inuit*, **37,** 103-125. (2013).
