# Supplementary material for: Zinc isotopes from archaeological bones provide reliable trophic level information for marine mammals
Source: Commun Biol. 2021 Jun 3;4:683. doi: 10.1038/s42003-021-02212-z (PMC8175341; doi:10.1038/s42003-021-02212-z)
Supplement: Supplementary file 3 — Description of Supplementary Files [file 42003_2021_2212_MOESM3_ESM.pdf]

Description of Additional Supplementary Files

File name: Supplementary Data 1

Description: $\delta^{13}C$, $\delta^{15}N$ and $\delta^{66}Zn$ dataset used in this study. Also included is the collagen (yield) weight % of carbon (wt. % C) and nitrogen (wt. % N), the collagen atomic carbon:nitrogen ratio (C/N), the bone mineral $\delta^{67}Zn$ and $\delta^{68}Zn$ values and Zn concentrations [Zn]. This dataset includes bone samples for which collagen $\delta^{13}C$ and $\delta^{15}N$ values were already reported elsewhere[4,15,30,31] as well as one site for which $\delta^{66}Zn$ was already reported[15] (sheet 1 in the accompanying .xlsx file).

File name: Supplementary Data 2

Description: $\delta^{66}Zn$ values for samples and reference material dissolved using different dissolution methods (Supplementary Methods 2.1, Supplementary Figure 2, 3). $\delta^{66}Zn$ values for samples and differences in $\delta^{66}Zn$ of bone material resampled and dissolved using the different dissolution methods ($\Delta^{66}Zn$; sheet 1 in the *accompanying* .xlsx file). $\delta^{66}Zn$ values for reference materials using the different dissolution methods (sheet 2 in the accompanying .xlsx file).

File name: Supplementary Data 3

Description: Results for post-hoc Tukey pair-wise comparisons between sites (indicated by their Borden code) for $\delta^{13}C$, $\delta^{15}N$ and $\delta^{66}Zn$ values of P. hispida (sheet 1-3 in the accompanying .xlsx file).
