# Supplementary material for: Zinc isotopes from archaeological bones provide reliable trophic level information for marine mammals
Source: Commun Biol. 2021 Jun 3;4:683. doi: 10.1038/s42003-021-02212-z (PMC8175341; doi:10.1038/s42003-021-02212-z)
Supplement: Supplementary file 7 — Reporting Summary [file 42003_2021_2212_MOESM7_ESM.pdf]

nature research

Corresponding author(s): Jeremy McCormack

Last updated by author(s): Apr 28, 2021

Reporting Summary

Nature Research wishes to improve the reproducibility of the work that we publish. This form provides structure for consistency and transparency in reporting. For further information on Nature Research policies, see our Editorial Policies and the Editorial Policy Checklist.

Statistics

For all statistical analyses, confirm that the following items are present in the figure legend, table legend, main text, or Methods section.

n/a	Confirmed	
☐	☒	The exact sample size (n) for each experimental group/condition, given as a discrete number and unit of measurement
☐	☒	A statement on whether measurements were taken from distinct samples or whether the same sample was measured repeatedly
☐	☒	The statistical test(s) used AND whether they are one- or two-sided<br>*Only common tests should be described solely by name; describe more complex techniques in the Methods section.*
☐	☒	A description of all covariates tested
☐	☒	A description of any assumptions or corrections, such as tests of normality and adjustment for multiple comparisons
☐	☒	A full description of the statistical parameters including central tendency (e.g. means) or other basic estimates (e.g. regression coefficient) AND variation (e.g. standard deviation) or associated estimates of uncertainty (e.g. confidence intervals)
☐	☒	For null hypothesis testing, the test statistic (e.g. F, t, r) with confidence intervals, effect sizes, degrees of freedom and P value noted<br>*Give P values as exact values whenever suitable.*
☒	☐	For Bayesian analysis, information on the choice of priors and Markov chain Monte Carlo settings
☒	☐	For hierarchical and complex designs, identification of the appropriate level for tests and full reporting of outcomes
☒	☐	Estimates of effect sizes (e.g. Cohen's d, Pearson's r), indicating how they were calculated

Our web collection on statistics for biologists contains articles on many of the points above.

Software and code

Policy information about availability of computer code

Data collection	n/a
Data analysis	All statistical analyses were conducted using the free program R software

For manuscripts utilizing custom algorithms or software that are central to the research but not yet described in published literature, software must be made available to editors and reviewers. We strongly encourage code deposition in a community repository (e.g. GitHub). See the Nature Research guidelines for submitting code & software for further information.

Data

Policy information about availability of data

All manuscripts must include a data availability statement. This statement should provide the following information, where applicable:

- Accession codes, unique identifiers, or web links for publicly available datasets
- A list of figures that have associated raw data
- A description of any restrictions on data availability

All data generated or analysed during this study are included in this published article (and its supplementary information files).

Field-specific reporting

Ecological, evolutionary & environmental sciences study design

All studies must disclose on these points even when the disclosure is negative.

Study description	Zinc isotopes as ecological proxies
Research sample	Bone samples from Arctic archaeological sites. Taxa analysed: Pusa hispida, Ursus maritimus, Pagophilus groenlandicus, Delphinapterus leucas, Odobenus rosmarus
Sampling strategy	Samples were chosen to achieve a high geographic spacing
Data collection	Mass spectrometry: Jeremy McCormack, Paul Szpak
Timing and spatial scale	n/a
Data exclusions	All data are reported. A single P. hispida specimen from Little Cornwallis Island (reference 13) was excluded from the statistical analysis. It was singled-out as an extreme zinc isotopy outlier lying more than 3 times the interquartile range above the third quartile, both within-site and for the whole P. hispida dataset.
Reproducibility	n/a
Randomization	n/a
Blinding	n/a

Did the study involve field work? ☐ Yes ☒ No

Reporting for specific materials, systems and methods

We require information from authors about some types of materials, experimental systems and methods used in many studies. Here, indicate whether each material, system or method listed is relevant to your study. If you are not sure if a list item applies to your research, read the appropriate section before selecting a response.

Materials & experimental systems

n/a	Involved in the study	
☒	☐	Antibodies
☒	☐	Eukaryotic cell lines
☐	☒	Palaeontology and archaeology
☒	☐	Animals and other organisms
☒	☐	Human research participants
☒	☐	Clinical data
☒	☐	Dual use research of concern

Methods

n/a	Involved in the study	
☒	☐	ChIP-seq
☒	☐	Flow cytometry
☒	☐	MRI-based neuroimaging

Palaeontology and Archaeology

Specimen provenance	Provenance information for the samples is provided in the supplementary material. Permission to conduct isotopic analyses on specimen from the Canadian Museum of History was granted on August 7, 2014. Permission to conduct isotopic analyses on specimen from the Avataq Cultural Institute was granted on November 14, 2013. Permission to conduct isotopic analyses on specimen from the Prince of Wales Northern Heritage Centre was granted on March 21, 2014.
Specimen deposition	No new specimens were excavated and deposited into collections as part of this research.
Dating methods	No new dates were obtained as part of this research. References for previously published dates are provided in the supplementary material.

☒ Tick this box to confirm that the raw and calibrated dates are available in the paper or in Supplementary Information.

Ethics oversight	Ethical approval was not required for this study as it did not involve human remains or culturally modified objects (i.e. artifacts).

Note that full information on the approval of the study protocol must also be provided in the manuscript.
